# Digital twins in nuclear medicine: A proposition of a modular pipeline for dosimetry protocol optimization in molecular radiotherapy

**DOI:** 10.1016/j.csbj.2025.08.027

**Published:** 2025-08-27

**Authors:** N. Sinsoilliez, B. Magnier, B. Piron, M. Bardiès, S. Janaqi, V. Boudousq

**Affiliations:** a EuroMov - Digital Health in Motion, IMT - MINES ALES, Université de Montpellier, France; b Institut de Recherche en Cancérologie de Montpellier (IRCM), Équipe Labellisée Ligue Contre le Cancer, INSERM U1194, Université de Montpellier, Institut régional du Cancer de Montpellier (ICM), Montpellier, France; c Nuclear Medicine Department, Institut régional du Cancer de Montpellier (ICM), Montpellier, France; d Nuclear Medicine Department, (CHRU Nîmes) Centre Hospitalier Régional Universitaire de Nîmes, France

**Keywords:** Digital twin, Dosimetry, Nuclear medicine, PRRT, PSMA, MCRPC

## Abstract

**Introduction:** Digital twins (DTs) are emerging tools for simulating and optimizing therapeutic protocols in personalized nuclear medicine. In this paper, we present a modular pipeline for constructing patient-specific DTs aimed at assessing and improving dosimetry protocols in PRRT such as L177u−PSMA therapy. **Materials & Methods:** The pipeline integrates three components: (i) an anatomical DT, generated by registering patient CT scans with an anthropomorphic model; (ii) a functional DT, based on a physiologically-based pharmacokinetic (PBPK) model created in SimBiology; and (iii) a virtual clinical trial module using GATE to simulate particle transport, image simulation, and absorbed dose distribution. Validation metrics include SSIM and DICE for registration quality, and MSE for PBPK model fitting with clinical quantification data. **Results** The anatomical DT module has been successfully implemented and validated on clinical data, demonstrating its ability to generate realistic, patient-morphed phantom for image-based dosimetry. The modular design allows for individual validation and reuse of each component, enabling stepwise development and integration. This architecture offers a strong foundation for evaluating dosimetry protocols and allowing multi-center standardization efforts in the future. **Conclusion:** This pipeline introduces a modular and adaptable DT framework to support protocol optimization in radionuclide therapy. As validation progresses, it holds strong potential for future use as a predictive tool for absorbed dose estimation prior to therapy, enabling safer and more effective personalized treatment planning.

## 1 Introduction

Peptide Receptor Radionuclide Therapy (PRRT) has emerged as an effective treatment option for metastatic castration-resistant prostate cancer (mCRPC), allowing for targeted irradiation of metastatic lesions throughout the body using radiopharmaceuticals such as L177u−PSMA [20]. However, current L177u−PSMA protocols follow a “one-size-fits-all” approach (7.4 GBq for 6 cycles), which has been increasingly questioned, and the benefits of personalized treatment for the patient have gained recognition [9]. Such strategies could help to optimize treatment on a per-patient basis, thereby enhancing the likelihood of successful tumor control and limited toxicity. 

To support such individualized approaches, patient-specific dosimetry plays a major role in tailoring the administered activity and treatment schedule by calculating the absorbed dose delivered to the organs and tissues. A dosimetry protocol is a multi-step process involving several interdisciplinary components, each with distinct trade-offs in terms of accuracy, complexity and reproducibility, and sources of errors and uncertainty, which should be thoroughly examined to determine the most appropriate option for ensuring optimal dosimetry.

While commercial solutions such as Hermes Voxel Dosimetry^1^ and MIM SurePlan™ MRT,^2^ and research-oriented solutions like OpenDose3D [7] are available, no consensus has yet been reached within the community on how to apply these protocols [12]. Cross-center and individual validation of each protocol remain major challenges [16], [21].

Virtual Clinical Trials (VCTs) provide a promising solution by enabling numerical experiments that simulate realistic treatment conditions without exposing patients to radiation or burdening clinical resources. However, such simulations must reflect biological and clinical realism to be useful. Additionally, virtual trials offer full control over all the parameters influencing the tests at each stage.

In this context, the concept of a Digital Twin (DT), a digital replica of a specific patient integrating anatomical and functional data, offers a structured and controllable environment to systematically test dosimetry protocols. By combining this concept with virtual trials, one can create realistic, parameterized simulations with known ground truth to benchmark different approaches.

This paper presents a modular pipeline for constructing a DT tailored to PRRT, integrating anatomically realistic patient-morphed phantoms and Physiologically Based PharmacoKinetic (PBPK) modeling as well as particle transport simulations. While some modules remain under development (e.g., PBPK model fitting), the overall framework lays the groundwork for a digital platform aimed at protocols optimization. Ultimately, this platform could be further developed into a tool for predicting absorbed doses as soon as the pre-therapy phase, allowing dosimetry-based therapeutic optimization of the treatment.

## 2 Materials & methods

### 2.1 Description of the proposed pipeline

**Fig. 1:**
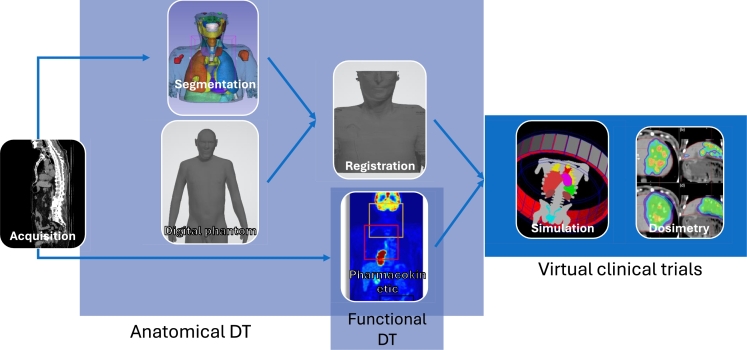
The proposed DT creation pipeline combines an anatomical DT and a functional DT to enable virtual clinical trials aimed at assessing and optimizing dosimetry protocols in nuclear medicine.

As illustrated in Fig. 1, the proposed DT creation method is structured into four main modules: data acquisition, anatomical DT, functional DT, and integration within VCTs.

The modular architecture enables the independent development, testing, and validation of each component within the overall pipeline, thereby improving reproducibility and robustness. This approach also supports focused updates or optimizations without impacting the entire system.

**Fig. 2:**
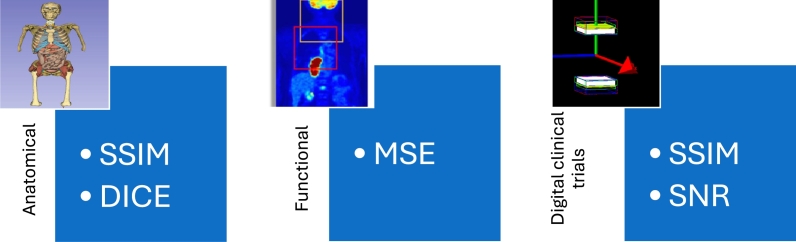
The SSIM and DICE are used to assess the quality of the “patient-morphed” phantom segmentation. The MSE serves as the error function to be minimized when fitting the PBPK model to clinical data. Finally, the SSIM and SNR are used to validate the DT's ability to replicate reality by comparing the clinical images with the images simulated by the virtual clinical trials.

As described below and detailed by the flowchart in Fig. 2, each module is individually validated to ensure the DT accurately reproduces clinical imaging and treatment conditions. Once validated, the DT can serve as a control platform to assess and optimize dosimetry protocols. By modifying selected parameters, one can evaluate their impact on therapeutic outcomes within a fully digital environment.

### 2.2 Acquisition process

This study focuses on patients with prostatic neoplasia treated with L177u−PSMA. Data were acquired in the nuclear medicine department of *CHU de Nîmes*, France, in accordance with IRB authorization n°23.12.04. Each patient receives a pre-therapy PET scan using G68a−PSMA followed by 6 therapeutic cycles of L177u−PSMA. This standardized clinical pathway ensures consistency across patient data and supports comparative analysis.

Quantitative SPECT/CT imaging was performed at 5, 48, and 168 hours post-injection (p.i.) using a GE Discovery NM/CT 670 gamma camera with MEGP collimator. Each scan includes three fields of view (ENT, thorax, abdomen/pelvis) and a low-dose CT for attenuation correction. PET imaging was acquired using GE OMNI Legend or Discovery 710 systems, with 4 to 5 acquisition steps of 2 minutes each, followed by a CT scan.

Additionally, the acquisition protocol was optimized for accurate image quantification, which is essential for PBPK model calibration and subsequent integration into the simulation pipeline. All images were pre-processed for segmentation, quantification, and simulations.

### 2.3 Anatomical model

The anatomical DT module begins with an automatic segmentation of the organs and structures of the CT images from the SPECT/CT and PET/CT acquisitions. We used “Annotate” software^3^ developed by TheraPanacea and previously validated for use in external radiotherapy [5]. Annotate was chosen for its ability to effectively segment both the salivary and lacrymal glands, organs that are sensitive to toxicity during L177u−PSMA therapy.

An XCAT 2 anthropomorphic model [19] was registered with the segmented data to create a “patient-morphed” digital model as described by Carter et al. [6]. The XCAT phantom offers anatomical realism, flexibility, and widespread validation in medical imaging research. Additionally, its anatomical library improves patient-specific matching even before the registration by covering a broad anatomical variability.

The XCAT is generated with the same image characteristics (resolution, number of images, etc.) as the patient's CT. This improves compatibility and minimizes discrepancies during the registration. The output data are converted to DICOM format, facilitating their integration into simulations and analysis.

Inspired by Fu et al. [11], registration is carried out using the wide range of registration methods (Rigid, Elastic, SyN, etc.) of the ANTs library [2]. Evaluation metrics include DICE coefficient and Structural Similarity Index Measure (SSIM) as described by [23]. Multiple registration methods and protocols (whole-body vs. per-organ) are tested, and the best-performing configuration is selected. Visual inspection completes the quality check.

### 2.4 Functional model

The functional component of the DT is a PBPK model specifically adapted to the treatment under investigation. Our work is based on the model developed by Kletting et al. [18] for treatments involving PSMA-labeled radiopharmaceuticals, which was modified for the purposes of our study. A major strength of this model is its ability to simultaneously represent diagnostic and therapeutic radiopharmaceutical, while accounting for the competition between free PSMA receptors and those occupied by labeled or unlabeled PSMA.

The model consists of two interconnected systems representing labeled PSMA and unlabeled PSMA. They share the same physiological distribution, differentiated by the radionuclide half-life. All major organs are represented. The model incorporates Absorption, Distribution, Metabolism, and Excretion (ADME). Additionally, it accounts for exchanges between vascular and interstitial tissues, as well as the internalization and release in and off the cells.

The model was developed using SimBiology, a MATLAB module designed for pharmacokinetic modeling. SimBiology was selected for its extensive control over model construction and simulation, as well as its easy integration into more complex pipelines [22]. Most physiological parameter values implemented were sourced from literature [4].

Only “patient-specific” parameters are defined by the user: age, body weight, and height; volumes of key organs (liver, spleen, kidneys, parotid glands, sub-mandibular glands, and lacrymal glands) extracted from the patient's CT images and amounts of labeled/unlabeled PSMA administered derived from the specific activity and volume of the injected radiopharmaceutical. “Patient-specific” values of renal blood flow, the fraction of blood filtered, the density of PSMA receptors in the kidney and prostate, and the renal release rate must be fitted using experimental data. This parameters fitting allows for any variations from the population average, or even pathological conditions such as renal failure.

To validate the model, simulated activity-time curves are compared to the quantification of the experimental PET/SPECT images. Quantification is carried out using the “Activity” method of the OpenDose3D software.^4^ The fitting process serves both to optimize the simulation parameters and to validate the model by minimizing the MSE error function between the 2 time-activity curves.

Once fitted, the PBPK model gives the activity distribution in each compartment at any p.i. time *x*. The distribution is integrated into the anatomical DT to generate a realistic voxelized source image at p.i.time t+x, where the voxel values correspond to the radioactive activity in that structure. At this stage, the implementation of the PBPK module is still under refinement, and its full integration into the simulation pipeline is ongoing.

### 2.5 Virtual clinical trials

Once the anatomical and functional aspects of the DT have been defined, this last module conducts the VCTs. It aims to replicate the nuclear medicine treatment process as realistically as possible using the previously generated DT data.

Simulations were carried out *in-silico* using GATE 9.2, an open-source platform dedicated to the simulation of particles and their interactions in the medical field [13], [17].

The GE Discovery NM/CT 670 SPECT system was modeled in detail, replicating the detector geometry, motion, and acquisition settings used for the clinical data. Particles were simulated according to the radionuclide used in the treatment. Additionally, attenuation and scattering were also taken into account.

The DT data was implemented through GATE macros as voxelized anatomical and activity distribution images. To obtain the dosimetric ground truth of the DT, an “absorbed dose actor”, i.e. a direct measurement during the simulation was implemented.

Validation of the VCT module was carried out by comparing the clinical PET/SPECT images with those simulated through GATE under identical acquisition conditions. To assess the DT's ability to replicate clinical reality, the SSIM and SNR metrics are employed to quantitatively compare the clinically acquired and GATE simulated images.

Although the virtual clinical trials module has been fully implemented and integrated into the DT pipeline, it currently remains in a validation phase. As the PBPK model is still under development, the functional input required to drive the VCTs is not available yet. Consequently, while the anatomical and dosimetric simulation components are functional, the end-to-end generation of output results is not yet possible.

## 3 Discussion

The DT pipeline presented in this work proposes a modular and realistic framework for dosimetry protocol assessment based on patient-specific data. Although still under development and not yet experimentally validated, the pipeline offers several methodological and pedagogical advances for the optimization and personalization of treatment protocols by merging anatomical, functional and simulation aspects into one coherent framework.

### 3.1 Limitations and improvements

#### 3.1.1 Registration

A refinement in the choice of the registration methods, as well as the use of a “pre-morphed” XCAT with patient-specific characteristics, should be considered in order to improve the overall efficiency and feasibility of the pipeline.

The recent emergence of deep learning-based registration methods should be noted. Among these, the Transformer architecture, such as VoxelMorph [3]and hybrid U-Net/Transformer models like TransMorph [8] have demonstrated superior registration performance compared to conventional methods. It could be useful to study the integration of such methods within the pipeline by comparing the registration quality to the methods described above, in order to assess whether AI-based approaches can achieve an even more realistic anatomical DT.

Finally, automatic segmentation methods are continually improving, making it worthwhile to explore the possibility of generating high-quality segmentation-based “patient-specific” phantoms. To our knowledge, no-one has yet compared these 2 approaches in the context of VCTs. However, the inherent challenges associated with the automatic segmentation of lesions and other anatomical variations suggest that using a ‘patient-morphed phantom’ remains necessary. Further studies are therefore required to assess the potential of such anatomical models.

#### 3.1.2 Pharmacokinetic

Parameter fitting of the PBPK model is highly sensitive to the quantification of the clinical images. It relies on a protocol comprising several stages (segmentation, measurement of the camera sensitivity factor, fitting of time activity curves, etc.) and each of those can introduce errors and uncertainties. As an example, if the number of SPECT/CT images is limited, the reliability of the quantification is impacted which can significantly impact the accuracy of the functional DT.

Nevertheless, studies such as Hardiansyah et al. [14], Devasia et al. [10] or Jackson et al. [15] are addressing this issue, particularly by investigating the possibility of reconstructing time-activity curves from a single p.i. acquisition. In the long term, this approach could enable the construction of the DT based solely on the pre-therapy PET/CT image, thereby further reducing clinical constraints.

In addition, other measurements, such as activity counts from blood or urine samples, could be considered to support the fitting process. This alternative offers complementary reference data for fitting while imposing relatively low additional clinical burden.

#### 3.1.3 Virtual clinical trials

Although the DT is designed to replicate patient anatomy and treatment characteristics as realistically as possible, it is sensitive to variations in input data and model assumptions. Establishing a comprehensive sensitivity analysis framework will be essential to assess and control this variability.

### 3.2 General perspectives

Initially developed for L177u−PSMA treatments, the DT offers, through its flexibility and modularity, the possibility of adapting to other therapeutic treatments such as Y90−DOTATATE, I131 or A225c−PSMA by adjusting the PBPK model and simulation parameters to reflect compound-specific kinetics and imaging characteristics. This would enable the study of challenges associated with certain radiopharmaceuticals, such as the difficulties in achieving quantitative imaging with Ac225-PSMA.

### 3.3 Modularity as a central design principle

A key strength of the proposed DT lies in its modular architecture. Each stage of the pipeline is designed to operate independently and can be easily replaced, modified or extended. This also makes the DT highly adaptable to various clinical scenarios and research contexts by integrating additional modules, such as radiobiological or immunological response to treatment, as proposed by Abdollahi et al. [1].

In the long term, by further enhancing the DT's realism, it could potentially serve as a pre-therapy tool for prediction of absorbed dose and patient-specific optimization of the treatment.

### 3.4 Potential application scenarios

This DT was built to serve as a platform for the study of dosimetry protocols. The objective is to evaluate the strengths, limitations, and key characteristics of the various procedures that have emerged in recent years.

Using a user-defined ground truth, the results of different protocols can be evaluated. In the same way, the impact of various treatment parameters on absorbed dose calculation, such as the number of quantitative p.i. acquisitions can be assessed. This approach aims to support nuclear medicine departments in selecting the protocols best suited to their therapeutic practices and clinical workflow.

**Fig. 3:**
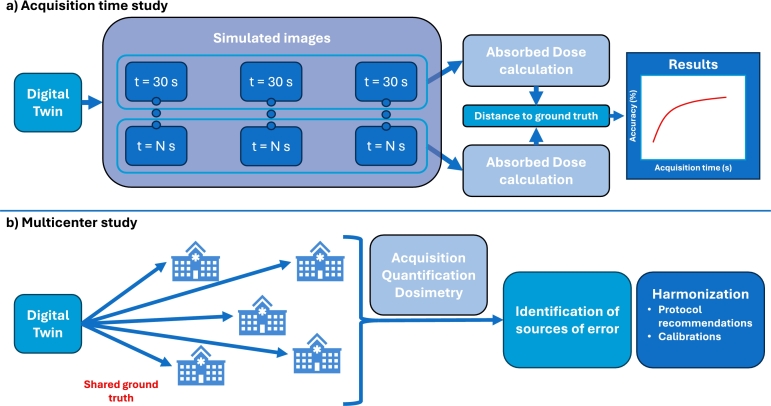
Potential applications of the DT framework: a) Acquisition time impact on absorbed dose calculation; b) Multicenter study using a shared ground truth, a step towards harmonizing clinical practices.

As illustrated in Fig. 3.a), one can study the impact of projection acquisition times on dosimetric results by modifying the acquisition times of the simulated images. Since acquisition time directly influences image quality, it also affects quantification accuracy and, consequently, imaging-based dosimetry. Similarly, the number of simulated p.i. acquisitions and the amount of activity administered to the patient can also be varied to assess their influence on dosimetric results. These investigations highlight the sensitivity of dosimetry protocols to variations in timing, administered activity, and other acquisition parameters. Using the DT for such studies offers a significant advantage: they can be carried out without clinical constraints or additional exposure to radiation to the patient, as all experiments are carried out solely through VCTs.

The DT can also serve as a test object for multicenter studies as shown in Fig. 3.b). By providing all participants with a shared ground truth, one can evaluate how variations in equipment, acquisition, quantification or dosimetry protocols across different centers influence the dosimetric calculations. This approach offers a controlled framework to identify methodological discrepancies and promote harmonization of clinical practices.

### 3.5 Usage constraints

The feasibility of this protocol highly depends on the availability of the data required to create the DT, which is not always guaranteed in routine clinical practice. In our case, complete patient data is required (medical data, anatomical data, blood tests, etc) as well as a CT scan, a pre-therapy PET scan and multiple SPECT/CT images p.i. However, this is not guaranteed in all nuclear medicine centers and departments. It is therefore essential to establish a routine for obtaining all the data required to create the DT before the start of therapy.

### 3.6 Future validation

While this study focuses on the design and methodological potential of the DT, future work will aim to validate the functional module and the global results of the pipeline in VCTs. This step will be critical for assessing the reliability of the DT and building trust for research and clinical use.

## 4 Conclusion

In the context of increasing interest in personalized approaches to molecular radiotherapy, especially for treatments such as L177u−PSMA, there is a growing need for tools that allow systematic assessments and optimization of dosimetric protocols. The main contribution of this study lies in the development of a DT conception pipeline capable of approximating anatomical and functional clinical reality in a controlled manner. This tool serves as a test bench for evaluating the influence of various treatment parameters, such as acquisition time and injected activity, on the results of the absorbed dose calculation.

This pipeline is based on 3 major pillars. Firstly, the creation of a “patient-morphed” digital model based on segmented CT images of the patient and registered with a standard digital model. This module is validated using SSIM and DICE metrics, ensuring that the anatomical DT reflects as close as possible the patient's characteristics. Secondly, simulation of the pharmacokinetic of the radiopharmaceutical within the patient's body using a PBPK model dedicated to L177u−PSMA. The parameters of this model are adjusted by minimizing the MSE between the simulations and the quantification of SPECT/CT and PET/CT images performed in routine clinical practice. Lastly, VCTs exploit the previous 2 pillars to generate a digital ground truth through simulation of particle transport, image simulation, and absorbed dose calculation.

The modular design of this pipeline allows the integration of complementary aspects related to treatments or patients, such as the radiobiological or immunological response. This flexibility enables the DT to be enriched according to the needs of each study.

Consequently, the possibility of extending the use of DT to absorbed dose prediction in the pre-therapy phase is by far the most interesting. Being able to simulate several different treatment protocols to determine which will optimize the delivery of absorbed dose to cancerous lesions while minimizing toxicity on healthy tissue would be a real advance in the field of nuclear medicine and personalized medicine.

## CRediT authorship contribution statement

**N. Sinsoilliez:** Writing – original draft, Validation, Methodology, Investigation, Formal analysis, Data curation, Conceptualization. **B. Magnier:** Writing – review & editing, Supervision. **B. Piron:** Writing – review & editing, Supervision. **M. Bardiès:** Writing – review & editing, Supervision. **S. Janaqi:** Writing – review & editing, Supervision. **V. Boudousq:** Writing – review & editing, Supervision.

## Ethical approval

This study was approved by the Ethics Committee of CHRU Nîmes under the IRB authorization n°23.12.04. Informed consent was obtained from all participants.

## Use of generative AI

During the preparation of this work, the authors used ChatGPT (OpenAI) to assist with language refinement. After using this tool, the authors reviewed and edited the content as needed and take full responsibility for the content of the publication.

## Funding

This research did not receive any specific grant from funding agencies in the public, commercial, or not-for-profit sectors.

## Declaration of Competing Interest

The authors declare the following financial interests/personal relationships which may be considered as potential competing interests:

Manuel Bardiès supervises a PhD Student sponsored by DOSIsoft.

Manuel Bardiès is a consultant for Clario.

## Footnotes

1 https://www.hermesmedical.com/our-software/dosimetry/voxel-dosimetry/.

2 http://mimsoftware.com/theranostics/sureplan-mrt.

3 https://www.therapanacea.eu/our-products/annotate/.

4 https://gitlab.com/opendose/opendose3d/-/blob/develop/User_Manual.md?ref_type=heads.
